# Mapping Traumatic Axonal Injury Using Diffusion Tensor Imaging: Correlations with Functional Outcome

**DOI:** 10.1371/journal.pone.0019214

**Published:** 2011-05-04

**Authors:** Virginia Newcombe, Doris Chatfield, Joanne Outtrim, Sarah Vowler, Anne Manktelow, Justin Cross, Daniel Scoffings, Martin Coleman, Peter Hutchinson, Jonathan Coles, T. Adrian Carpenter, John Pickard, Guy Williams, David Menon

**Affiliations:** 1 University Division of Anaesthesia, University of Cambridge, Cambridge, United Kingdom; 2 Wolfson Brain Imaging Centre, Department of Clinical Neurosciences, University of Cambridge, Cambridge, United Kingdom; 3 Centre for Applied Medical Statistics, Department of Public Health and Primary Care, University of Cambridge, Cambridge, United Kingdom; 4 Department of Radiology, University of Cambridge, Addenbrooke's Hospital, Cambridge, United Kingdom; 5 Academic Neurosurgery Unit, Department of Clinical Neurosciences, University of Cambridge, Cambridge, United Kingdom; University of California San Francisco, United States of America

## Abstract

**Background:**

Traumatic brain injury is a major cause of morbidity and mortality worldwide. Ameliorating the neurocognitive and physical deficits that accompany traumatic brain injury would be of substantial benefit, but the mechanisms that underlie them are poorly characterized. This study aimed to use diffusion tensor imaging to relate clinical outcome to the burden of white matter injury.

**Methodology/Principal Findings:**

Sixty-eight patients, categorized by the Glasgow Outcome Score, underwent magnetic resonance imaging at a median of 11.8 months (range 6.6 months to 3.7 years) years post injury. Control data were obtained from 36 age-matched healthy volunteers. Mean fractional anisotropy, apparent diffusion coefficient (ADC), and eigenvalues were obtained for regions of interest commonly affected in traumatic brain injury. In a subset of patients where conventional magnetic resonance imaging was completely normal, diffusion tensor imaging was able to detect clear abnormalities. Significant trends of increasing ADC with worse outcome were noted in all regions of interest. In the white matter regions of interest worse clinical outcome corresponded with significant trends of decreasing fractional anisotropy.

**Conclusions/Significance:**

This study found that clinical outcome was related to the burden of white matter injury, quantified by diffusivity parameters late after traumatic brain injury. These differences were seen even in patients with the best outcomes and patients in whom conventional magnetic resonance imaging was normal, suggesting that diffusion tensor imaging can detect subtle injury missed by other techniques. An improved *in vivo* understanding of the pathology of traumatic brain injury, including its distribution and extent, may enhance outcome evaluation and help to provide a mechanistic basis for deficits that remain unexplained by other approaches.

## Introduction

Traumatic brain injury (TBI) is a major cause of morbidity and mortality worldwide. The extent and severity of traumatic brain injury is greatly underestimated by X-ray computed tomography (CT) and conventional magnetic resonance imaging (MRI), which often correlate poorly with functional outcome [1], [2], [3]. Indeed, some patients may have no visible abnormalities and yet experience significant neurocognitive sequelae post-TBI. These neurocognitive outcomes are disabling for the individual, and expensive for society [4], [5], [6]. Although ameliorating these deficits would be of substantial benefit the mechanisms that underlie them are poorly characterized.

There is an increasing belief that many of the cognitive deficits following TBI may be the consequence of traumatic axonal injury (TAI), which may be subtle and is poorly quantified with conventional imaging techniques. MRI with diffusion tensor imaging (DTI) characterizes the diffusion of water molecules in tissue environments, which is influenced by the microstructural organization of tissues and their constituent cells, and can provide unique insights into pathophysiology, particularly in white matter. The diffusion tensor can be used to represent the magnitude of water diffusion (quantified as the apparent diffusion coefficient, ADC), whether such diffusion is directionally non-uniform (anisotropy), and the orientation of that direction (eigenvectors/eigenvalues). Indeed, previous studies have used the technique in TBI, and typically found consistent reductions in fractional anisotropy (FA) in classical areas affected by TAI, even when conventional MRI showed no lesion. These regions include the subcortical white matter in the frontal and temporal regions, splenium of the corpus callosum, posterior limb of the internal capsule, and the cerebral peduncles [7], [8], [9], [10], [11], [12], [13]. FA has also been noted to be decreased in other regions, including the cingulum [9] and fornix [14], and ROIs that encompass the entire white matter but show no lesion [15].

Despite these accumulating data on DTI in TBI, previous studies have reported on small numbers of patients and/or addressed a limited range of outcome categories. We wished to examine how clinical outcomes related to the burden of white matter injury, with outcomes ranging from the vegetative state to patients with no or minimal sequelae.

## Methods

### Ethics Statement

Ethical approval was obtained from the Cambridgeshire 2 Research Ethics Committee, and written informed consent, or written assent from next-of-kin where appropriate, were obtained in all cases in accordance with the Declaration of Helsinki.

Sixty-eight patients who had sustained TBI underwent MR imaging using a 3 Tesla Siemens Magnetom Total Imaging Matrix (TIM) Trio. Thirty-six controls (healthy volunteers) underwent an identical imaging protocol. This included DTI, 3D T1 weighted structural imaging (magnetization prepared rapid gradient echo; MPRAGE), a Fluid Attenuated Inversion Recovery (FLAIR) sequence, a gradient echo (GE) sequence, and a dual echo (proton density/T2) sequence. The DTI parameters were as follows; 12 non-collinear directions, 5 b values ranging from 338 to 1588 s/mm^2^, 5 b = 0 images, acquisition matrix size 96×96, field of view 192 mm×192 mm, 63 axial slices, 2 mm slice thickness, TR = 8300 ms, TE = 98 ms. All scans were visually inspected and four patients with translational head movement greater than 5 mm during the diffusion sequence were removed prior to data analysis. This left a dataset of 64 patients and 36 controls. All conventional images were inspected by two neuroradiologists (JC and DS), blinded to whether the images were from control subjects or patients with TBI, and to the outcome category of individual patients. The presence and location of lesions were noted. Subsequent creation of regions of interest (ROIs) took account of this information, and ensured that they did not include lesioned tissue, since blood products may cause signal dropout in DTI.

The DTI data underwent eddy current correction and FA, ADC and eigenvalue maps were created using the Oxford Centre for fMRI of the Brain's (FMRIB's) Diffusion Toolbox (http://www.fmrib.ox.ac.uk/fsl/). To aid coregistration the skull, and extracranial soft tissue were stripped from the MPRAGE images using the Brain Extraction Tool [16]. The diffusion weighted data were normalized using a two step approach. First, all patient and control MPRAGE images were coregistered to the MNI152 template using the vtkCISG normalized mutual information algorithm (http://www.image-registration.com). The b = 0 image was subsequently coregistered to the subject's own MPRAGE image. The transformation matrix normalizing the MPRAGE image was then applied to the b = 0 image. After each step, the data were visually inspected to exclude processing errors.

**Figure 1:**
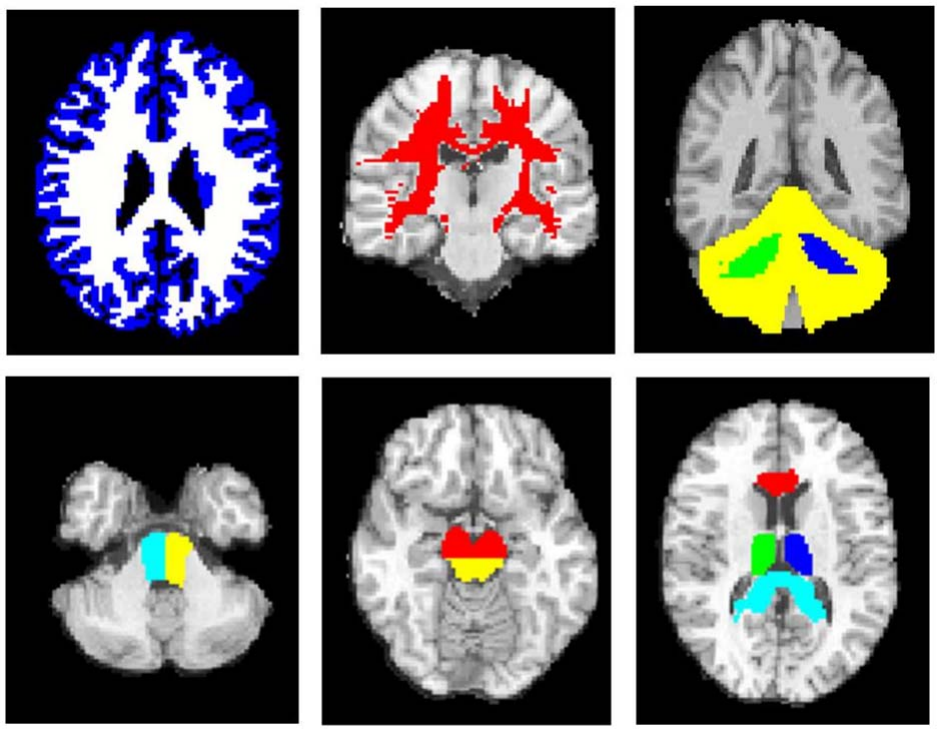
Examples of the regions of interest used. Top from left to right; whole brain grey matter (blue), whole brain white mater (white), the supratentorial white matter (red), right and left cerebellar peduncles (green and blue) and the cerebellar cortex (yellow). Bottom from left to right; right and left pons (light blue and yellow), dorsal (yellow) and ventral (red) midbrain, thalamus (green and blue), anterior corpus callosum (red) and posterior corpus callous (light blue).

ROIs, chosen due to their predilection for damage post TBI, were manually drawn using Analyze 7.0 (http://www.mayo.edu/bir) in MNI125 space using Colin27 [17] as a high resolution, high signal-to-noise template, and included the corpus callosum (genu and splenium), thalamus, midbrain, pons, cerebellar peduncles, and cerebellar cortex (Figure 1). Each subject's own MPRAGE image was segmented to make whole brain white matter (WBWM) and whole brain grey matter (WBGM) masks, using Automated Segmentation Tool (FAST; FMRIB, Oxford, UK) [18], which was coregistered to normalized space. A supratentorial WM (SWM) ROI was created by subtracting the cortical grey matter and infratentorial WM from the WBWM mask. All coregistered images were visually inspected to ensure that ROIs corresponded to the regions specified, and any lesions identified by radiological reporting were manually removed using Analyze 7.0.The mean ADC, FA and eigenvalues for the different ROIs were calculated using in-house software (written by GBW). Axial diffusivity was defined as the major eigenvalue (λ1) and radial diffusivity as the average of the two minor eigenvalues ((λ2+λ3)/2).

Patients were categorized into groups using the Glasgow Outcome Scale (GOS), which uses six simple questions in the domains of physical, neuropsychological and social disability, and is the most widely used outcome measure post TBI [19], [20]. It has been shown to have good reliability and validity across many different populations groups [21], [22]. This classification is generally undertaken at least 6 months post injury, with the categories as follows; 1: dead, 2: vegetative state, 3: severe disability (conscious but dependent), 4: moderate disability (disabled but independent) and 5: good recovery [19]. The GOS is often dichotomized into Unfavorable (GOS categories 1 to 3) and Favorable (GOS categories 4 and 5) outcomes. Although it has been criticized as being a somewhat crude scale, it has the advantage of being relatively easy to obtain, and is generalizable across patient populations. The majority of patients in our study were at, or past, the six month time point, but one patient (with a clinical diagnosis of VS) was imaged three months post injury. The upper three categories of the GOS (GOS 3 to 5) may be subdivided, creating an eight-point scale or the extended GOS (eGOS) [22], [23]. As data for GOS was available for all patients, but only 90% had GOSE available, GOS was used as the main clinical outcome variable.

Statistical analyses were conducted using SPSS14.0 (http://www.spss.com) and graphs were produced using StatView (SAS Institute Inc., 1998). Due to the small sample size in some groups, all analyses using the GOS categories were performed using non-parametric statistics. Mann-Whitney U (MHU) was used for unpaired tests and the Wilcoxon signed rank test for paired comparisons. Since use of the Spearman test is inappropriate for ordinal categories of less than ten, the Jonckheere-Terpstra Test was used to test for trends in DTI parameters with outcome category. The main analysis involved a total of 38 comparisons for trend between outcome and DTI parameters, and p values were accepted as significant if they were corrected for multiple comparisons using the Bonferroni correction (p<0.0013). For other analyses, p<0.05 was accepted as significant.

## Results

The patient demographic details are shown in Table 1. No evidence of differences in age or injury to MRI interval was found between any of the groups. No systematic differences in DTI parameters were observed between left *vs.* right sided ROIs, or between patients with large and small structural abnormalities (using a 1.5 cm lesion diameter as a cut off). In order to increase statistical power, data in each of these categories were pooled for intergroup statistical comparisons.

**Table 1:** Summary of the demographic and clinical characteristics of controls and patients.

	Controls	TBI patients			
	(n = 36)	GOS 5(n = 21)	GOS 4(n = 20)	GOS 3(n = 16)	GOS 2(n = 7)
**Age at scan (years) (mean, range)**	38 (24 to 70)	32 (18 to 59)	38 (20 to 60)	38.8 (17 to 63)	39 (21 to 67)
**Injury to MRI interval (days) (median, range)**		306 (172 to 1252)	387 (174 to 1341)	373 (192 to 1130)	198 (105 to 681)
**Gender (number (percentage))**					
Male	27 (75)	14 (66.7)	14 (70)	8 (50)	5 (71)
Female	9 (25)	7 (33.3)	6 (30)	8 (50)	2 (29)
**Cause of Injury (number (percentage))**					
Motor Vehicle Collision		17 (81)	14 (70)	10 (62.5)	3 (42.9)
Assault		1 (4.8)	2 (10)	1 (6.3)	2 (28.6)
Fall		3 (14.3)	4 (20)	5 (31.3)	2 (28.6)

GOS = Glasgow Outcome Score at time of scan [19]; 1 = death, 2 = persistent vegetative state, 3 = severe disability, 4 = moderate disability, 5 = good recovery.

The TBI patients were divided into groups based on Glasgow Outcome Score (GOS) at the time of imaging.

**Figure 2:**
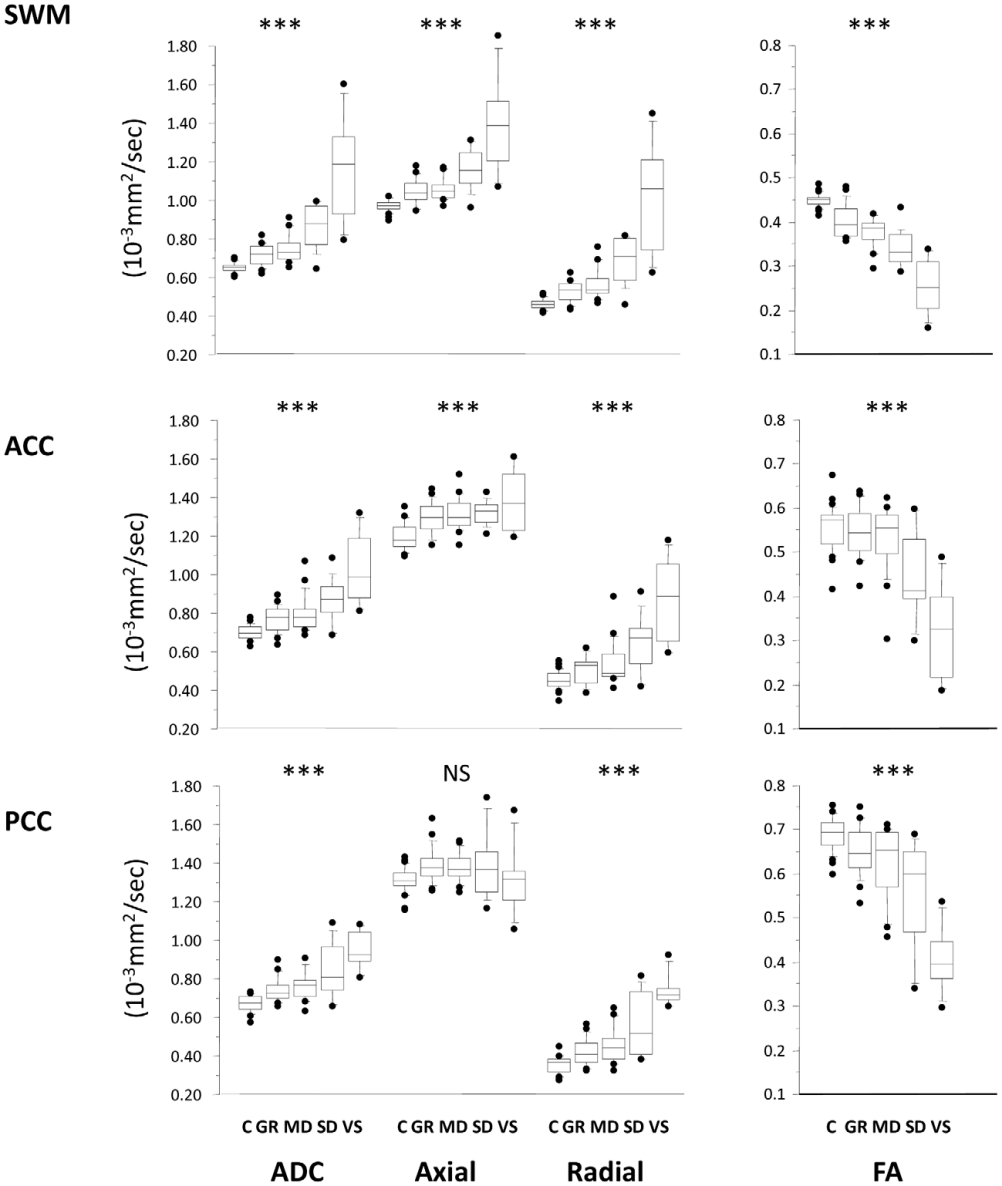
Trends for the ADC (left), axial, radial diffusivity and FA (right) for supratentorial white matter ROIs. The central lines in the boxes denote the median values, the upper and lower edges the 75^th^ and 25^th^ percentiles, the error bars the 90^th^ and 10^th^ percentiles and the closed circles the data outside these percentiles. *** p<0.0001; NS, non-significant. SWM: supratentorial white matter, ACC: anterior corpus callosum, PCC: posterior corpus callosum. C = controls, GR = good recovery, MD = moderate disability, SD = severe disability, VS = vegetative state.

Clinical outcome showed an inverse trend with ADC in all ROIs (Figures 2–5, Supplementary Table S1). A corresponding trend for decrease in FA with worsening outcome was found in the predominantly WM ROIs. These changes appear to be a consequence not only of an increase in radial diffusivity, but also increases in axial diffusivity in the SWM, ACC, pons, thalamus, and WBGM. No correlation was found between time from injury and DTI parameters in any ROI. There was good sensitivity and specificity for all ROIs to distinguish between patients with favourable versus unfavourable recovery as evidenced by the area under the receiver operating curve (ROC). This discrimination was mainly driven by differences between GOS 2 and GOS 3, and to a smaller (but still significant) extent by differences between GOS 3 and GOS 4 (Table 2). However, apart from differences between GOS 2 and 3, DTI (in general) poorly differentiated between adjacent GOS categories.

**Figure 3 pone-0019214-g003:**
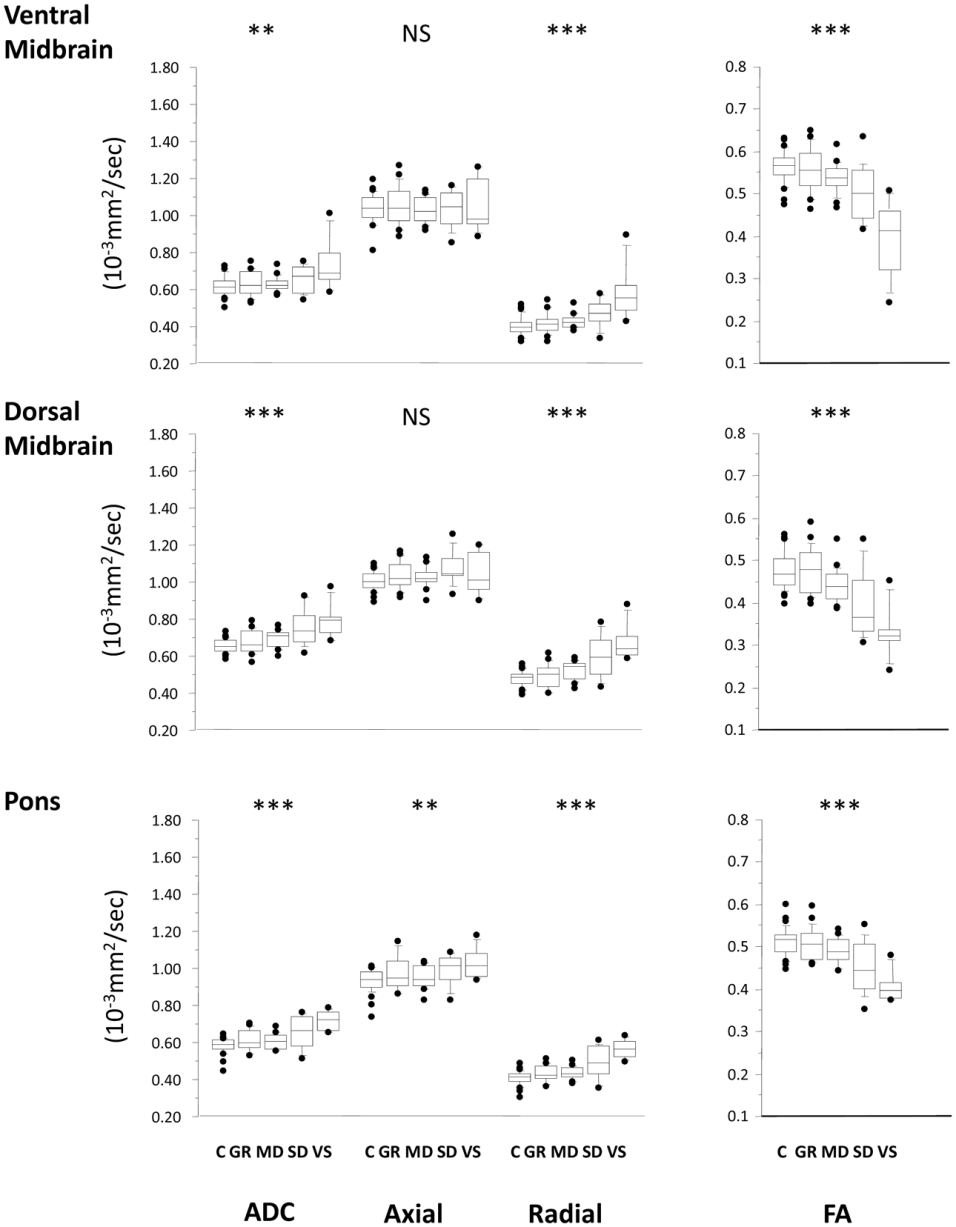
Trends for the ADC (left), axial, radial diffusivity and FA (right) for brainstem ROIs. The central lines in the boxes denote the median values, the upper and lower edges the 75^th^ and 25^th^ percentiles, the error bars the 90^th^ and 10^th^ percentiles and the closed circles the data outside these percentiles. *** p<0.0001; NS, non-significant. SWM: supratentorial white matter, ACC: anterior corpus callosum, PCC: posterior corpus callosum. C = controls, GR = good recovery, MD = moderate disability, SD = severe disability, VS = vegetative state.

**Figure 4 pone-0019214-g004:**
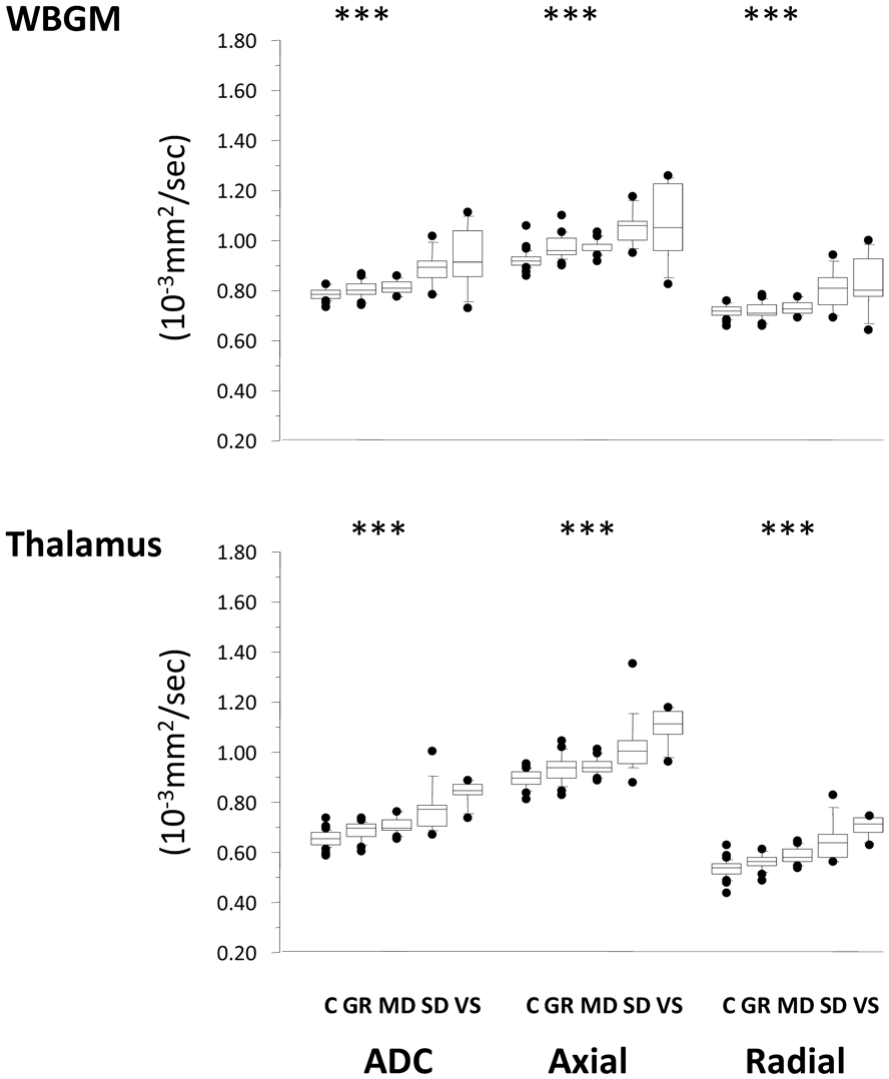
Trends in ADC, axial and radial diffusivity for the ROIs compromised predominantly of grey matter. C = controls, GR = good recovery, MD = moderate disability, SD = severe disability, VS = vegetative state. * p<0·0013; **<0.001; *** p<0.0001; NS, non-significant.

**Figure 5:**
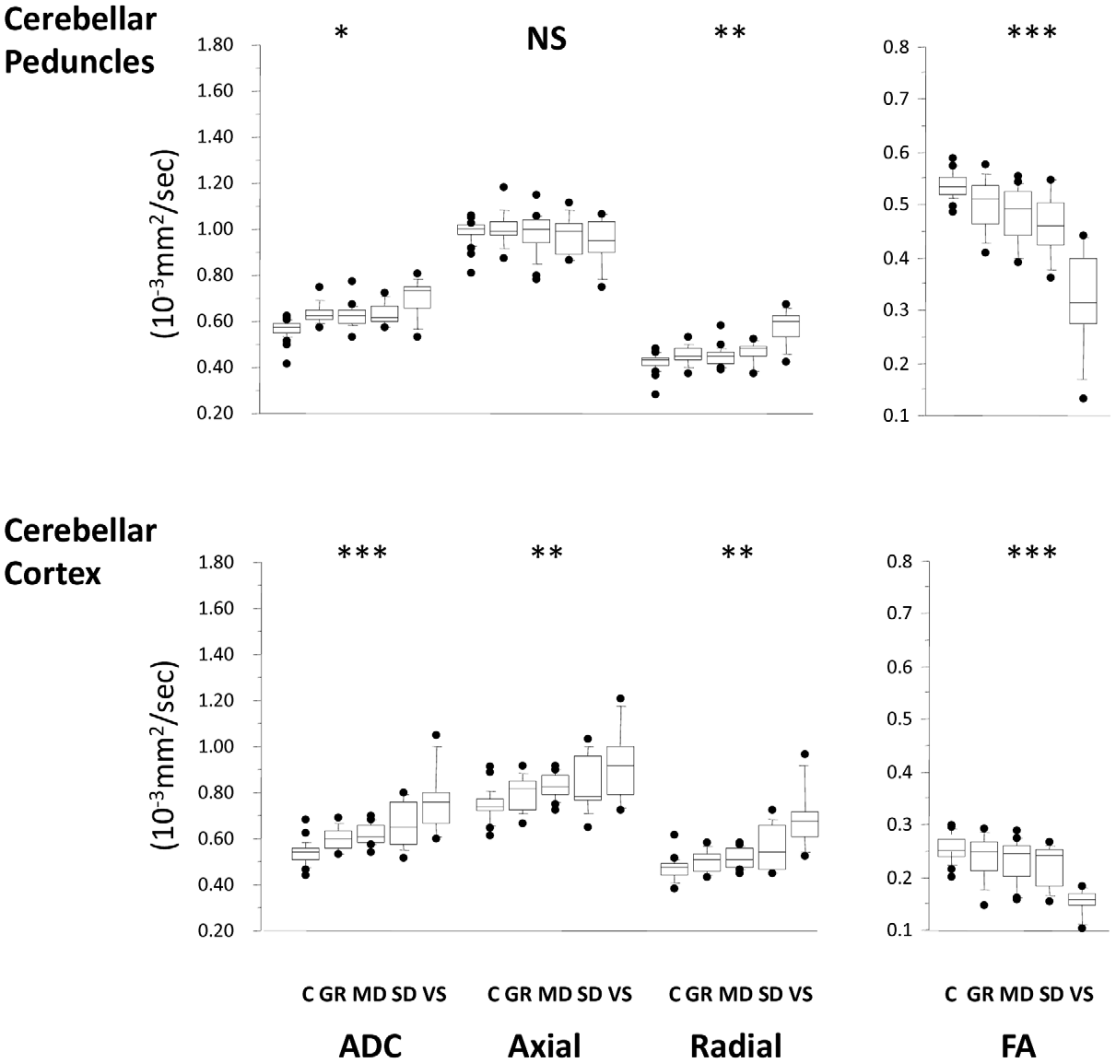
Trends in ADC, axial and radial diffusivity for the cerebellar peduncles and the cerebellar cortex. C = controls, GR = good recovery, MD = moderate disability, SD = severe disability, VS = vegetative state. * p<0·0013; ** <0.001; *** p<0.0001; NS, non-significant.

**Table 2:** Comparison of adjoining GOS categories as well as the receiver operating curve characteristics for patients with favourable versus unfavourable outcomes.

	FA						ADC					
Regions of Interest	GOS 5 Vs GOS 4	GOS 4 Vs GOS 3	GOS 3 Vs GOS 2	Favourable Vs Unfavourable(P value)	Area under the ROC curve(95% CI)	P value	GOS 5 Vs GOS 4	GOS 4 Vs GOS 3	GOS 3 Vs GOS 2	Favourable Vs Unfavourable(P value)	Area under the ROC curve(95% CI)	P value
Supratentorial white matter	0.565	0.002	0.010	0.000	0.877(0.773 to 0.980)	0.000	0.433	0.004	0.014	0.000	0.896(0.798 to 0.994)	0.000
Anterior corpus callosum	0.261	0.017	0.040	0.000	0.840(0.721 to 0.959)	0.000	0.448	0.066	0.046	0.000	0.806(0.676 to 0.935)	0.000
Posterior corpus callosum	0.530	0.186	0.001	0.000	0.797(0.671 to 0.923)	0.000	0.403	0.503	0.016	0.002	0.750(0.603 to 0.897)	0.002
Ventral midbrain	0.979	0.279	0.016	0.000	0.796(0.66 to 0.932)	0.000	0.754	0.744	0.012	0.009	0.705(0.549 to 0.860)	0.009
Dorsal midbrain	0.094	0.175	0.030	0.000	0.775(0.621 to 0.93)	0.000	0.200	0.503	0.016	0.001	0.757(0.626 to 0.888)	0.001
Pons	0.619	0.264	0.002	0.000	0.813(0.670 to 0.956)	0.000	0.937	0.440	0.002	0.001	0.722(0.569 to 0.874)	0.005
WBGM	0.374	0.279	0.010	0.001	0.762(0.628 to 0.896)	0.001	0.638	0.020	0.434	0.004	0.724(0.558 to 0.890)	0.004
Thalamus	0.855	0.250	0.794	0.416	0.635(0.474 to 0.796)	0.087	0.958	0.311	0.010	0.000	0.879(0.780 to 0.978)	0.000
Cerebellar peduncles	0.350	0.231	0.001	0.000	0.823(0.697 to 0.950)	0.000	0.248	0.452	0.023	0.052	0.654(0.487 to 0.820)	0.049
Cerebellar cortex	0.762	0.707	0.002	0.002	0.740(0.603 to 0.877)	0.002	0.425	0.321	0.824	0.064	0.645(0.478 to 0.811)	0.064

GOS Glasgow Outcome Score, ROC = receiver operating curve, CI = confidence intervals.

**Figure 6:**
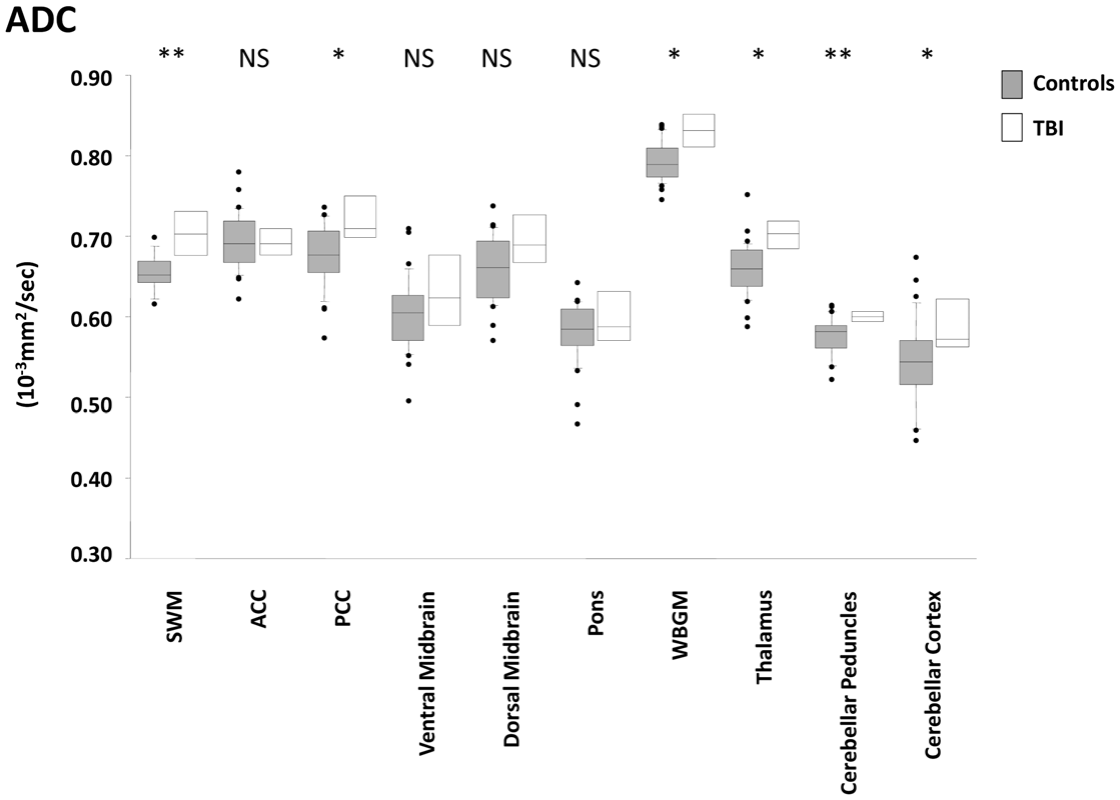
Comparison of ADCs for the ROIs in the control group (n = 20) versus a group of four patients (TBI) (one GOS 3, two GOS 4, one GOS 5) who had normal appearing conventional sequences. Control data are shown in grey, and patients in white. For FA only SWM was significantly lower in this subset of patients. SWM: supratentorial white matter, ACC: anterior corpus callosum, PCC: posterior corpus callosum, WBGM: whole brain grey matter. The p-value pertains to a Mann-Whitney U (exact) test between the two groups. * p<0·05; ** <0.01; NS, non-significant.

A small subgroup of four patients had their conventional MR sequences reported as normal by both neuroradiologists. Their clinical characteristics are shown in Table 3. Despite normal conventional radiology, only one of these was clinically classified as having achieved a Good Recovery (GOS 5). In spite of their normal appearing structural MRI scans, this exhibited a significant decrease in FA in the SWM and increased ADC in the SWM, PCC, WBGM, thalamus, cerebellar peduncles and the cerebellar cortex (Figure 6).

**Table 3:** Clinical Characteristics of the four patients with normal appearing conventional MR sequences.

Patient	Cause of Injury	Age at Injury	Gender	Injury to MRI interval (days)	GCS at ictus	GOS
1	RTA	37	Male	1130	15	3
2	RTA	46	Female	2342	13	4
3	RTA	46	Male	677	15	4
4	RTA	27	Female	1097	14	5

## Discussion

To our knowledge, this study is the first to use DTI to investigate the full spectrum of outcome of TBI patients in the chronic phase post injury, ranging from the vegetative state to minimal or no disability. We show gradations of DTI abnormality in a broad range of ROIs, with patients with worse outcomes having lower FA and higher ADCs. An eigenvalue analysis of DTI data suggested that the changes in FA were associated with increases in both radial and axial diffusivity. These findings support the inclusion of DTI in the portfolio of imaging tools used to characterize the burden of insult following TBI.

Previous studies have found little correlation between CT and/or conventional MR sequences on one hand, and cognitive and functional outcomes on the other [2], [3]. We found that quantitative DTI was sensitive in detecting damaged tissue, and, perhaps more importantly, that these imaging measures correlated with a full range of outcomes post-TBI. The detection of these changes is of particular interest in cohorts of patients who have no abnormalities detected on CT or conventional MRI. In these cases DTI may provide the only available means of documenting the anatomical substrate for late neuropsychological deficits post-TBI. Multiple mechanisms may underlie these late changes, including demyelination, axonal disconnection, astrogliois, and damage to intracellular cytoskeleton and neurofilaments [24]. Indeed, in a small subset of patients that were reported as having normal MRI despite having functional deficits, abnormal DTI parameters were documented even in ROIs that were not selected based on patient symptomatology (Figure 5). These data suggest that the functional deficits observed in TBI survivors may be the consequence of damage to integrated neuronal systems, rather than lesions at focal injury sites.

Significant differences in DTI parameters in the central WM, WBGM, corpus callosum (anterior and posterior) and the thalamus were found in comparisons between all patients groups. However, the midbrain and pons ROIs were only significantly different to controls in patients in the poorest outcome groups (GOS 2 and 3). This may indicate that damage to these areas is particularly important in determining whether a patient develops permanent impairments in consciousness or not. Indeed, brainstem lesions have previously been associated with unfavorable outcomes in TBI [3], [25], [26].

The increase in diffusivity in both radial and axial directions may be expected in grey matter regions like the WBGM and thalamus, where cellular necrosis may result in less restricted diffusion. However, we also noted this finding in predominantly white matter ROIs, such as the central WM, pons, and the anterior corpus callosum. Such changes would not be explained by simple demyelination, which would only predictably increase radial diffusivity [27]. However, change in axonal microstructure might also reduce the restriction of water diffusion along the long axis of the axon. Alternatively, this finding may imply a change in the dominant cell type contributing to the signal, with axonal bundles being replaced by astrocytes and/or microglia, with increased diffusion in all directions.

The patients studied here encompassed a wide range of disability. It is difficult to find robust cognitive tasks and functional measures that are applicable across such a broad spectrum of patients, who range from the vegetative state, to those able to return to work with minimal or no impairment. In this context, the GOS has several advantages: it characterizes the entire spectrum of TBI outcomes, is easily obtained and reproducible, and is widely used. These attributes make our results more easily applicable and interpreted in the context of other cohorts of TBI patients. However, despite these advantages of the GOS, the lack of refinement in describing some outcomes may be a disadvantage. For example, in GOS category 3, patients in the minimally conscious state (patients who exhibit inconsistent, but reproducible responsiveness; MCS) are grouped with patients who, while unable to live independently, are cognitively far less disabled. Arguably, MCS patients are clinically more similar to the VS patients than those at the higher end of GOS 3, but the framework of the GOS does not permit such reallocation. In any event, a reanalysis with the MCS and VS patients grouped together produced similar results.

**Figure 7:**
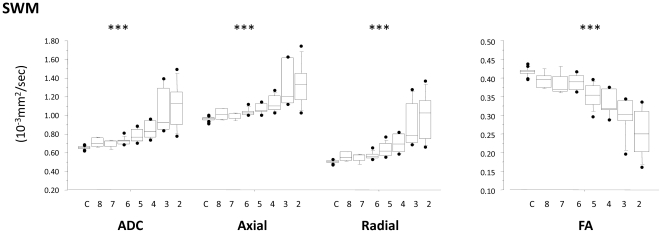
Results for the supratentorial white matter ROI as an example of the groups categorized into the Glasgow Outcome Score Extended. 2 to 8 represent GOSe categories 2 to 8 and C is the control group.

One approach to a more refined outcome classification would be to use the extended Glasgow outcome scale (GOSe). In 90% of our patients we had outcome data that allowed such categorization, and a reanalysis with patients categorized in this way did not materially change our inferences about the association between DTI parameters and clinical outcome (see Figure 7 for an example). The fact that GOSe outcome data could not be calculated for a significant minority lead us to use the GOS as the basis for our definitive analysis. Further refinement of the relationship between imaging and outcome may be possible within each GOS category by using outcome variables applicable to that particular category (e.g. formal neuropsychological testing in patients in the GOS 5 category (good outcomes)). Such an approach will require data from a larger sample of patients.

The patients were also studied at varying time points after TBI, but, except for one patient who was diagnosed to be in VS, had a minimum interval between injury and imaging of approximately six months. It is possible that continuing clinical recovery may have resulted in some reclassification of functional outcome in some patients. However, many studies in TBI use a follow up time point of six months post-injury, recognising that a substantial proportion of clinical recovery occurs by this time point. Notwithstanding this, a future study that used uniform (and potentially serial) late follow up and imaging would produce useful corroboration of our findings. In addition, larger studies, particularly involving patients with little damage on conventional imaging, may allow more subtle differences in outcome and neurocognitive functioning to be correlated with DTI parameters.

Finally, our demonstration of pervasive DTI abnormalities in the cerebellum which scale with functional outcome reflect a growing understanding that cerebellar lesions may be important in defining TBI outcome. In a perceptive position paper, Ghajar and Ivry summarized the evidence for abnormalities of cerebellar function contributing to cognitive deficits in TBI [28]. They suggested that the deficits in TBI may be due to a dysfunction in the “predictive brain state”, part of which could be attributed to cerebellar dysfunction. Further, they suggested that DTI might provide insights into the subtle abnormalities in key loops that connect the frontal lobes, basal ganglia and the cerebellum. Our data provide some evidence to support their hypotheses.

We have shown that clinical outcome relates to the burden of white matter injury, as quantified by diffusivity parameters in patients in the chronic phase post TBI. These DTI abnormalities are seen even in patients with the best outcomes, and in patients with normal conventional MRI, suggesting that they can detect subtle injury that is missed by other approaches. Our data thus provide a basis for including DTI in evaluating TBI outcome, while providing a mechanistic basis for deficits that remained unexplained by other approaches.

## Supporting Information

Table S1 Median (interquartile range) for diffusivity parameters for the Central WM, WBGM, and corpus callosum (genu and splenium) by subject group.
(DOC)
