# Supplementary material for: Mapping Traumatic Axonal Injury Using Diffusion Tensor Imaging: Correlations with Functional Outcome
Source: PLoS One. 2011 May 4;6(5):e19214. doi: 10.1371/journal.pone.0019214 (PMC3087728; doi:10.1371/journal.pone.0019214)
Supplement: Table S1 — Median (interquartile range) for diffusivity parameters for the Central WM, WBGM, and corpus callosum (genu and splenium) by subject group. (DOC) [file pone.0019214.s001.doc]

**Supporting Information**

**Table S1:** **Median (interquartile range) for diffusivity parameters for the Central WM, WBGM, and corpus callosum (genu and splenium) by subject group.**

The significance level for the Jonckheere-Terpstra Test for Trend is shown.

|  |  | **Controls** | **GR** | **MD** | **SD** | **VS** | **p-value (Test for Trend)** |
| --- | --- | --- | --- | --- | --- | --- | --- |
| **SWM** | **FA** | 0.42 (0.40 to 0.42) | 0.39 (0.36 to 0.43) | 0.39 (0.36 to 0.40) | 0.32 (0.29 to 0.37) | 0.25 (0.20 to 0.32) | <0.0001 |
|  | **ADC** | 0.66 (0.65 to 0.67) | 0.72 (0.68 to 0.76) | 0.73 (0.70 to 0.77) | 0.87 (0.77 to 0.95) | 1.13 (0.90 to 1.28) | <0.0001 |
|  | **Axial** | 0.97 (0.95 to 0.98) | 1.03 (1.00 to 1.08) | 1.04 (1.01 to 1.07) | 1.13 (1.07 to 1.22) | 1.33 (1.16 to 1.45) | <0.0001 |
|  | **Radial** | 0.50 (0.49 to 0.52) | 0.56 (0.52 to 0.60) | 0.57 (0.55 to 0.62) | 0.74 (0.62 to 0.81) | 1.03 (0.74 to 1.19) | <0.0001 |
| **Corpus Callosum** | **FA** | 0.57 (0.51 to 0.59) | 0.54 (0.49 to 0.59) | 0.56 (0.50 to 0.59) | 0.41 (0.36 to 0.53) | 0.33 (0.21 to 0.42) | <0.0001 |
| **Genu** | **ADC** | 0.69 (0.67 to 0.72) | 0.78 (0.71 to 0.82) | 0.77 (0.72 to 0.82) | 0.88 (0.80 to 0.97) | 0.99 (0.85 to 1.19) | <0.0001 |
|  | **Axial** | 1.17 (1.14 to 1.24) | 1.30 (1.23 to 1.36) | 1.29 (1.25 to 1.36) | 1.31 (1.27 to 1.35) | 1.37 (1.20 to 1.57) | <0.0001 |
|  | **Radial** | 0.44 (0.42 to 0.48) | 0.53 (0.44 to 0.57) | 0.49 (0.46 to 0.58) | 0.69 (0.54 to 0.78) | 0.88 (0.61 to 1.06) | <0.0001 |
| **Corpus Callosum** | **FA** | 0.69 (0.66 to 0.71) | 0.65 (0.60 to 0.69) | 0.64 (0.57 to 0.69) | 0.54 (0.37 to 0.65) | 0.37 (0.30 to 0.46) | <0.0001 |
| **Splenium** | **ADC** | 0.68 (0.65 to 0.71) | 0.72 (0.70 to 0.79) | 0.77 (0.71 to 0.81) | 0.82 (0.75 to 0.99) | 0.92 (0.88 to 1.07) | <0.0001 |
|  | **Axial** | 1.32 (1.27 to 1.36) | 1.38 (1.33 to 1.45) | 1.37 (1.33 to 1.45) | 1.37 (1.26 to 1.46) | 1.31 (1.22 to 1.41) | 0.023 |
|  | **Radial** | 0.36 (0.32 to 0.39) | 0.41 (0.37 to 0.49) | 0.45 (0.38 to 0.51) | 0.57 (0.41 to 0.78) | 0.74 (0.68 to 0.78) | <0.0001 |
| **WBGM** | **FA** | 0.17 (0.17 to 0.18) | 0.22 (0.20 (0.24) | 0.21 (0.18 to 0.22) | 0.19 (0.17 to 0.21) | 0.17 (0.13 to 0.20) | 0.119 |
|  | **ADC** | 0.79 (0.77 to 0.81) | 0.81 (0.79 to 0.84) | 0.82 (0.80 to 0.84) | 0.90 (0.84 to 0.92) | 0.91 (0.76 to 0.95) | <0.0001 |
|  | **Axial** | 0.92 (0.91 to 0.94) | 0.98 (0.95 to 1.04) | 0.99 (0.96 to 1.00) | 1.06 (1.00 to 1.08) | 1.05 (0.86 to 1.16) | <0.0001 |
|  | **Radial** | 0.73 (0.71 to 0.75) | 0.72 (0.70 to 0.76) | 0.74 (0.72 to 0.76) | 0.82 (0.74 to 0.85) | 0.84 (0.71 to 0.87) | <0.0001 |
| **Thalamus** | **FA** | 0.34 (0.33 to 0.36) | 0.33 (0.32 to 0.36) | 0.33 (0.31 to 0.35) | 0.33 (0.41 to 0.36) | 0.32 (0.29 to 0.35) | 0.018 |
|  | **ADC** | 0.66 (0.63 to 0.68) | 0.70 (0.67 to 0.72) | 0.70 (0.69 to 0.73) | 0.77 (0.71 to 0.85) | 0.85 (0.83 to 0.88) | <0.0001 |
|  | **Axial** | 0.90 (0.87 to 0.93) | 0.94 (0.90 to 0.98) | 0.94 (0.93 to 0.96) | 1.02 (0.96 to 1.10) | 1.12 (1.07 to 1.17) | <0.0001 |
|  | **Radial** | 0.54 (0.52 to 0.56) | 0.57 (0.55 to 0.59) | 0.58 (0.56 to 0,62) | 0.64 (0.59 to 0.72) | 0.72 (0.67 to 0.74) | <0.0001 |
| **Ventral**  **Midbrain** | **FA** | 0.56 (0.54 to 08.58) | 0.55 (0.51 to 0.59) | 0.54 (0.52 to 0.56) | 0.47 (0.42 to 0.55) | 0.39 (0.36 to 0.48) | <0.0001 |
|  | **ADC** | 0.61 (0.57 to 0.64) | 0.64 (0.57 to 0.70) | 0.62 (0.60 to 0.65) | 0.68 (0.59 to 0.73) | 0.69 (0.63 to 0.80) | 0.0001 |
|  | **Axial** | 1.02 (0.98 to 1.09) | 1.04 (0.97 to 1.14) | 1.03 (0.96 to 1.09) | 1.05 (0.97 to 1.12) | 0.97 (0.89 to 1.25) | 0.907 |
|  | **Radial** | 0.40 (0.37 to 0.42) | 0.41 (0.37 to 0.47) | 0.42 (0.40 to 0.44) | 0.48 (0.43 to 0.56) | 0.55 (0.50 to 0.63) | <0.0001 |
| **Dorsal**  **Midbrain** | **FA** | 0.47 (0.44 to 0.50) | 0.47 (0.42 to 0.52) | 0.44 (0.41 to 0.47) | 0.36 (0.33 to 0.50) | 0.31 (0.25 to 0.34) | <0.0001 |
|  | **ADC** | 0.66 (0.62 to 0.69) | 0.67 (0.63 to 0.74) | 0.70 (0.66 to 0.74) | 0.74 (0.69 to 0.82) | 0.80 (0.75 to 0.83) | <0.0001 |
|  | **Axial** | 1.01 (0.98 to 1.04) | 1.03 (0.99 to 1.12) | 1.03 (1.01 to 1.07) | 1.05 (1.01 to 1.18) | 1.04 (1.00 to 1.20) | 0.025 |
|  | **Radial** | 0.49 (0.45 to 0.51) | 0.52 (0.44 to 0.55) | 0.55 (0.49 to 0.56) | 0.59 (0.52 to 0.69) | 0.69 (0.60 to 0.73) | <0.0001 |
| **Pons** | **FA** | 0.51 (0.48 to 0.52) | 0.50 (0.47 to 0,53) | 0.49 (0.47 to 0.51) | 0.43 (0.40 to 0.50) | 0.39 (0.37 to 0.41) | <0.0001 |
|  | **ADC** | 0.58 (0.55 to 0.61) | 0.59 (0.57 to 0.67) | 0.59 (0.56 to 0.64) | 0.66 (0.59 to 0.74) | 0.71 (0.65 to 0.76) | <0.0001 |
|  | **Axial** | 0.92 (0.89 to 0.97) | 0.95 (0.90 to 1.04) | 0.94 (0.90 to 1.01) | 1.01 (0.91 to 1.05) | 1.01 (0.95 to 1.08) | 0.004 |
|  | **Radial** | 0.41 (0.39 to 0.43) | 0.41 (0.40 to 0.48) | 0.42 (0.40 to 0.46) | 0.50 (0.43 to 0.58) | 0.56 (0.50 to 0.61) | <0.0001 |
| **Cerebellar**  **Peduncles** | **FA** | 0.53 (0.52 to 0.55) | 0.51 (0.46 to 0.54) | 0.49 (0.44 to 0.53) | 0.46 (0.40 to 0.52) | 0.32 (0.25 to 0.38) | <0.0001 |
|  | **ADC** | 0.58 (0.54 to 0.59) | 0.64 (0.62 to 0.65) | 0.63 (0.59 to 0.69) | 0.62 (0.60 to 0.67) | 0.71 (0.63 to 0.76) | 0.011 |
|  | **Axial** | 1.00 (0.99 to 1.02) | 0.99 (0.97 to 1.04) | 1.00 (0.94 to 1.04) | 0.99 (0.89 to 1.03) | 0.98 (0.86 to 1.05) | 0.214 |
|  | **Radial** | 0.41 (.39 to 0.42) | 0.43 (0.41 to 0.45) | 0.45 (0.43 to 0.48) | 0.48 (0.43 to 0.49) | 0.58 (0.48 to 0.63) | 0.0006 |
| **Cerebellar**  **Cortex** | **FA** | 0.27 (0.25 to 0.30) | 0.26 (0.22 to 0.28) | 0.26 (0.21 to 0.27) | 0.25 (0.18 to 0.27) | 0.16 (0.13 to 0.18) |  |
|  | **ADC** | 0.55 (0.53 to 0.56) | 0.60 (0.55 to 0.64) | 0.60 (0.57 to 0.68) | 0.63 (0.58 to 0.77) | 0.68 (0.56 to 0.76) | <0.0001 |
|  | **Axial** | 0.73 (0.71 to 0.75) | 0.80 (0.72 to 0.85) | 0.79 (0.71 to 0.87) | 0.82 (0.76 to 0.98) | 0.81 (0.70 to 0.90) | 0.0008 |
|  | **Radial** | 0.48 (0.46 to 0.49) | 0.51 (0.46 to 0.54) | 0.50 (0.48 to 0.69) | 0.55 (0.50 to 0.66) | 0.62 (0.46 to 0.69) | 0.0005 |

Units for ADC, axial and radial diffusivity: (x10^-3^mm^2^/sec), GOS = Glasgow Outcome Score at time of scan[19]; GR = good recovery, MD = moderate disability, SD = severe disability, VS = vegetative state, WBWM: whole brain white matter, WBGM: whole brain grey matter
